# Underwater Noise Level Recordings from a Water Intake Pontoon and Possible Impacts on Yangtze Finless Porpoises in a Natural Reserve

**DOI:** 10.3390/ani12172183

**Published:** 2022-08-25

**Authors:** Wenfei Lu, Jianfeng Tong, Xianfeng Zhang, Bin Zhu, Weiwei Dong

**Affiliations:** 1 College of Marine Sciences, Shanghai Ocean University, Shanghai 201306, China; 2 Joint Laboratory for Monitoring and Conservation of Aquatic Living Resources in the Yangtze Estuary, Shanghai 201306, China; 3 National Distant-Water Fisheries Engineering Research Center, Shanghai 201306, China; 4 Institute of Hydrobiology, Chinese Academy of Sciences, Wuhan 430072, China; 5 Institute of Hydroecology, MWR&CAS, Wuhan 430079, China

**Keywords:** Yangtze finless porpoise, underwater noise, water intake pontoon, natural reserve

## Abstract

**Simple Summary:**

Relocation of endangered freshwater cetaceans in natural reserves is an effective conservation strategy to recover their populations. However, due to historical reasons, some natural reserves have facilities that cannot be removed, such as water intake pontoons that are important for residents nearby. These facilities generate underwater noise which may affect cetaceans. To investigate the potential impacts of underwater noise from water intake pontoons on freshwater cetaceans, this study measured such noise within a Yangtze finless porpoise (*Neophocaena asiaeorientalis*) nature reserve, analyzed its effects on porpoise behavior, vocalization, and hearing, and explored porpoise adaptation to the transient holding pen environment. This study provides a reference for assessing whether human facilities have impacts on freshwater cetaceans.

**Abstract:**

Underwater noise pollution caused by human activities may affect freshwater cetaceans to different degrees. To analyze the impacts of water intake pontoons on Yangtze finless porpoises (*Neophocaena asiaeorientalis*), this study collected underwater noise data from such a pontoon in a nature reserve, plotted the power spectral density of acoustic signals, and calculated the root mean square sound pressure levels and the magnitude of sound source levels. The 1/3-octave sound pressure level root mean square values at the transient holding pens were <18.0 kHz, 39.5−60.0 kHz, which were slightly higher than the Yangtze finless porpoise hearing threshold curve values and therefore could be perceived. However, the results indicated that the porpoises would not develop a temporary hearing threshold shift. Meanwhile, pontoon noise did not interfere with the porpoises’ high-frequency acoustic signal nor did it affect their echolocation; it significantly interfered with their low-frequency acoustic signal, however, and the mother–child communication of the finless porpoises was affected, but this effect was quickly compensated due to the limited space range of the holding pens. Through this study of Yangtze finless porpoises, this paper provides a reference for assessing whether human facilities have impacts on freshwater cetaceans.

## 1. Introduction

The Yangtze River finless porpoise (*Neophocaena asiaeorientalis*) is the only freshwater cetacean left in China’s Yangtze River Basin after the functional extinction of the baiji dolphin (*Lipotes vexillifer*) [1]. It is currently distributed only in the middle and lower reaches of the Yangtze River and its connections to Poyang and Dongting Lakes [2,3]. Many Yangtze finless porpoise habitats are severely damaged and the finless porpoise is facing the threat of a sharp decline in fish stocks [4,5]. The porpoise has been listed in the First Order of Protected Animals in China [6] and has also been listed as critically endangered on the IUCN Red List of Threatened Species since 2013 [7]. One of the main threats to porpoise survival is underwater noise pollution caused by human activities [3]. Underwater noise generated by ships and river crossings directly interferes with the hearing system of the porpoise, affecting communication, positioning, feeding, and other daily activities [8]. Therefore, strengthening research on underwater noise caused by water-related projects and analyzing the threat that it poses to the porpoise can help to protect them and improve the protection of other freshwater cetaceans.

Previous studies have been conducted to measure underwater noises from different human activities in the Yangtze River, which may affect Yangtze finless porpoises. Wang et al. [9] analyzed underwater noise from sand-mining vessels in Dongting Lake, whose sound source levels were 150−170 dB re 1 μPa and whose energy was mostly concentrated below 1 kHz. The Yangtze finless porpoise audiogram has two optimal sensitivity regions at frequencies of 45 and 108−128 kHz, with thresholds of 55 and 60 dB, respectively [10]. Underwater noise exceeded the hearing threshold of the Yangtze finless porpoise, which was theoretically sufficient to cause hearing damage. Ju et al. [11] studied the riprapping noise during the waterway adjustment activities of the lower Yangtze River and the northern branch of the Changzhou River, and found that the sound source level of riprapping noise was greater than 151 dB and that its energy was mainly concentrated in the low and middle frequencies (<20 kHz). The recorded 1/3-octave sound pressure level was higher than the hearing threshold of Yangtze finless porpoise at most frequencies. In combination with porpoise acoustic signal and auditory characteristics, it is believed that the riprapping noise may affect their natural habitat. Zhang et al. [12] recorded navigational noise from various types of large vessels in the navigable river section of the Yangtze River and Changzhou Beicha, finding that the main source levels were between 149.5 and 156.0 dB, mainly high-intensity low- and medium-frequency noise. This indicated that noise from large vessels may have a negative impact on acoustic communication and hearing among Yangtze finless porpoises, such as auditory masking.

Because the Yangtze finless porpoise is critically endangered, certain protective measures need to be taken. Relocation is an important initiative for their conservation [13,14], and it plays an important role in scientific research [6,15]. The Hewangmiao Yangtze finless porpoise reserve is located in the territory of Jianli City, Hubei Province, China, and the area has excellent geographical and natural environmental conditions [16]. It is an established reserve to which the Yangtze finless porpoise has been relocated. To implement the 2021 Yangtze finless porpoise natural ex situ conservation action plan of the Ministry of Agriculture and Rural Affairs of China, two holding pens were set up outside the management office pontoon of the reserve as a temporary housing and rescue site. Due to historical reasons, a water intake pontoon is about 12 m away from the holding pens. It pumps Yangtze River water through the pipeline to a water treatment plant for public use and therefore must operate all year round, generating continuous underwater noise. No study has been conducted on the underwater noise of the water intake pontoon. Therefore, it is important to investigate its impact on the Yangtze River finless porpoise. 

In this study, we measured underwater noise at different locations around the water intake pontoon, analyzed the noise effects on Yangtze finless porpoises in holding pens, and explored the adaptability of porpoises to the transient holding pens environment. This paper provides a reference for assessing whether human facilities have impacts on freshwater cetaceans. 

## 2. Materials and Methods

### 2.1. Study Site and Subject

**Figure 1:**
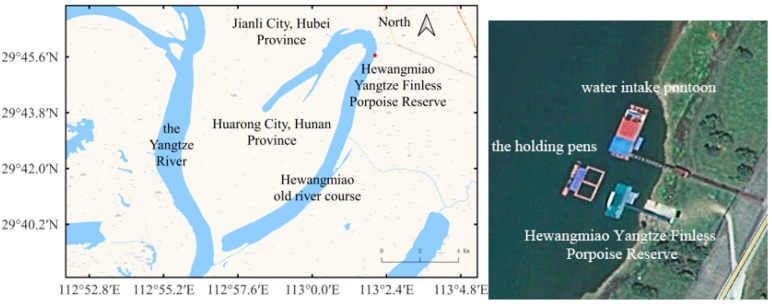
Study site: Hewangmiao Yangtze Finless Porpoise Reserve, China.

**Figure 2:**
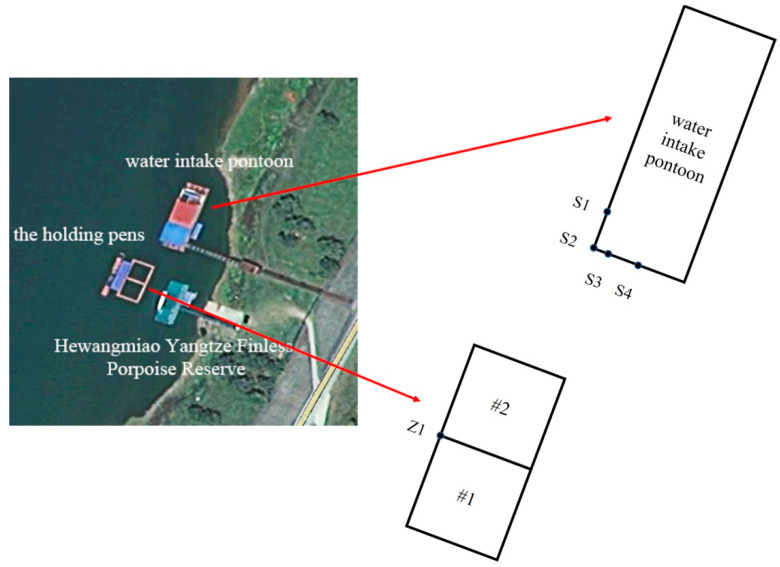
Sampling points (S1 to S4 were set up at the water intake pontoon; Z1 was set up at the holding pens).

The study area was the Hewangmiao Yangtze finless porpoise sanctuary in Jianli City, Hubei Province, China. Two 10 × 10 × 5 m^3^ Yangtze finless porpoise transient holding pens were set up side by side: the lower left one was labeled #1 and the upper right one was labeled #2 (Figure 1 and Figure 2). During the study, three male individuals were temporarily reared in holding pen #1, and three individuals, one male and two females, were temporarily reared in holding pen #2. All the porpoises were aged from 2 to 3 years.

The water intake pontoon is about 12 m away from the nearest holding pen. The pontoon is about 30 m long and 10 m wide. It works non-stop all year round, thus generating underwater noise.

### 2.2. Acoustic Data Collection

The underwater noise measurement was conducted on June 4 2021, next to the holding pens and with the pontoon 12 m away from holding pen #2. Four sampling points were set up at the water intake pontoon (Figure 2). S1 was the closest to the noise source motor. S2 was the closest point from the pontoon to the #2 holding pen. The distance between S1 and S2 was about 3 m. The distance between S3 and S2 was about 1 m. The distance between S4 and S2 was about 5.6 m. Sampling point Z1 was set up at the intersection of the two holding pens.

Acoustic recorders (SoundTrap ST300, Ocean Instruments, New Zealand) were used to collect underwater noise from the water intake pontoon at fixed points. The recorder was tied to a heavy object at one end to ensure that it could fall vertically into the water, and the other end was attached to the pontoon railing or a transient holding pen at a distance of about 2 m. After ensuring that there was no other human interference, the acoustic recorder was turned on by remote control. The recorder parameters were as follows: effective working frequency range: 20 Hz−60 kHz ± 3 dB; sensitivity: −176.7 dB re 1 V/μPa (high-gain gear); sampling rate: 144 kHz; analog-to-digital conversion bit count: 16 bits. There was no breeze or rain during the acoustic data collection, and no vessels passed, which excluded the influence of natural factors and other human activities on the sampling. Due to equipment limitations, only one recording equipment set could be used at a sampling point when acoustic data were being collected, so each sampling point was not conducted simultaneously. However, because only 10 min of sampling was done at each sampling point, there were no significant changes in hydroclimatic conditions and human disturbances, so the collection points could be considered simultaneous recordings.

### 2.3. Acoustic Data Analysis

The waveforms and spectrograms of the audio at each sampling point were viewed visually using RavenPro 1.6 software (The Cornell Lab of Ornithology, Ithaca, NY, USA), and data with higher signal-to-noise ratio were selected by comparing the waveforms [9]. Representative 5s audio files for each sampling point were selected.

A custom program was written using MATLAB software (MathWorks software, Natick, MA, USA) to calibrate and analyze the 5s audio files for each sampling point. The Hanning window (frame length 8192, window overlap 50%) was chosen to plot power spectral density (PSD) and sound pressure level curves, while performing a fast Fourier transform (FFT) [17]. The noise level at each sampling point was analyzed by combining the formulas to calculate the root mean square sound pressure level (SPL_rms_ in dB re 1 μPa), sound exposure level (SEL in dB re 1 μPa^2^·s), and source level (SL in dB re 1 μPa) of underwater noise generated during the operation of the pontoon motor [18,19,20]. 

In addition, the root mean square values of 1/3-octave sound pressure levels at different frequencies were calculated for the audio at each sampling point [21] and plotted as curves, which were subsequently compared with the porpoise hearing threshold curves.

### 2.4. Behavioral Observations

Behavioral observations were conducted during the transient captivity of Yangtze finless porpoises in the holding pens. The three males were moved into holding pen #1 on 26 April 2021, and the one male and two females were moved into holding pen #2 27 April 2021. All the finless porpoises were relocated to the open water of the reserve on 5 June 2021. After being placed in the pens, the respiration rate of the finless porpoises was recorded every 10 min for 5 min, and the swimming posture was closely observed. When the respiration rate was normal and the swimming posture was gentle, the interval time for recording the respiration rate was changed to 0.5, 1, and 2 h. The finless porpoises were first fed about 2 h after being placed in the pens. After the finless porpoises had eaten several times, they started to be fed regularly, four times per day. The daily mean respiration rate and food consumption were analyzed to evaluate the adaptivity of the finless porpoises to the holding pens environment.

## 3. Results

### 3.1. Comparison of Noise Intensity

The broadband (bandwidth of 20 Hz to 60 kHz) acoustic parameter measurements at each sampling point are summarized in Table 1. The SPL_rms_ of the holding pens was 108.4 dB. Because the underwater structure of the pontoon was not yet known, the sound from the motor may have been partially obscured. Therefore, the specific location of the sound source could not be inferred from the sound pressure level at the sampling points. According to the analysis, the estimated sound SL of underwater noise generated by the water intake pontoon motor during operation was greater than 149.5 dB.

### 3.2. Distribution of Noise Energy from the Water Intake Pontoon at Different Frequencies

**Figure 3:**
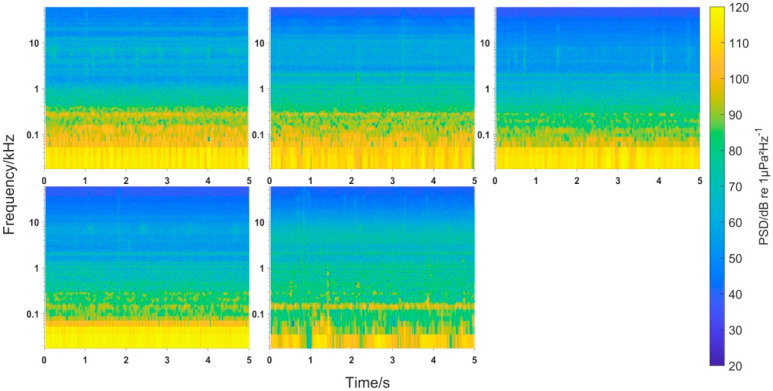
Power spectral densities of all sampling points (first row: S1, S2, S3; second row: S4, Z1; frequency range 20 Hz−60 kHz).

**Figure 4:**
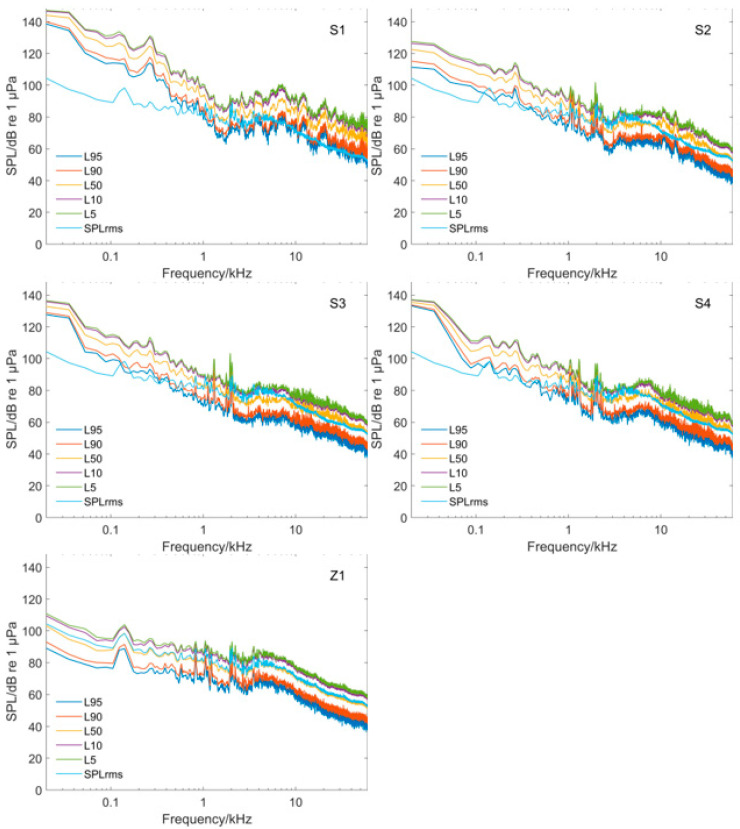
Probability distribution pattern of noise level at different frequencies of each sampling point (frequency range 20 Hz−60 kHz).

**Figure 5:**
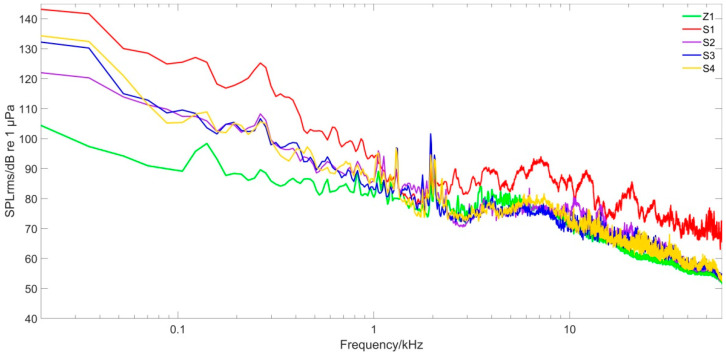
Comparison among SPL_rms_ of each sampling point (frequency range 20 Hz−60 kHz).

To study the underwater noise energy distribution of the water intake pontoon at different frequencies, the PSD was plotted for each sampling point (Figure 3). The probability density distribution and the SPL of the noise at different frequencies were plotted (Figure 4), where Ln is the cumulative percentage sound level. This meant that the sound pressure level exceeded Ln for n% of the time during the measurement period. In addition, the SPL_rms_ values for each sampling point were compared and analyzed (Figure 5).

The energy concentration range of each sampling point was mainly in the low- and middle-frequency range of less than 10 kHz, whereas the energy distribution was concentrated in the low-frequency part less than 100 Hz (Figure 3). Because sampling point Z1 at the transient holding pen was at a given distance from the sound source, its underwater noise intensity was lower than the other sampling points.

The underwater noise generated by the water intake pontoon was mainly high-intensity low-frequency noise (Figure 4). The SPL declined overall with increasing frequency, and there was no strong high-frequency component. Because sampling point S1 was closer to the sound source of the pontoon, the SPL could reach 143.5 dB and the SPL curve was higher than other sampling points with relative variability. The holding pen sample point Z1 had a maximum SPL of 106.0 dB. It was about 30 m away from the sound source so sound propagation through the water was somewhat reduced. The SPL at Z1 was 37.5 dB lower than that at S1, the closest sampling point to the water intake pontoon source, and the overall SPL curve was lower than that at other sampling points with less variability.

In terms of the SPL_rms_ at each frequency (Figure 5), the noise at sampling point Z1 ranged from 106.0 to 52.1 dB, and at sampling point S1 it ranged from 143.5 to 68.3 dB. The noise at the transient holding pens was reduced by 16.2−37.5 dB at each frequency compared with the noise at the water intake pontoon. The SPL significantly increased at frequencies less than 1 kHz.

### 3.3. Comparison of the 1/3-Octave Power Spectrum of Water Intake Pontoon Noise

**Figure 6:**
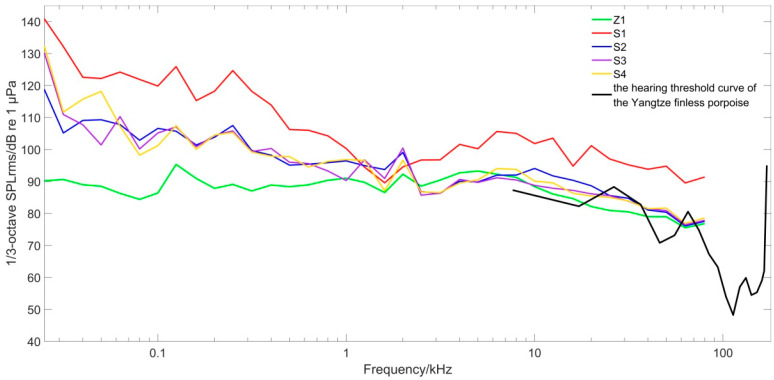
1/3-octave SPL_rms_ of each sampling point and audiogram of the Yangtze finless porpoise (frequency range 25 Hz−180 kHz; the porpoise audiogram is reproduced from Popov et al., 2005 [23], with the permission of the Acoustical Society of America).

Because the effective filter bandwidth of the mammalian auditory system is close to 1/3 octave [22], we calculated the 1/3-octave bandwidth SPL of the noise at each sampling point to analyze its possible effect on porpoise hearing (Figure 6).

Due to the close proximity of sampling point S1 to the water intake pontoon source, its 1/3-octave SPL_rms_ values were higher than those of other sampling points in all frequency ranges (except 1250−2000 Hz). The 1/3-octave underwater noise SPL_rms_ values at S2, S3, and S4 were similar. Respective values at Z1 were lower than those of other sampling points except for the frequency range of 2500−10,000 Hz. Z1 values were between 78.5 and 90.2 dB, and the noise at the sampling point of the water intake pontoon was between 91.0 and 140.8 dB. The 1/3-octave SPL_rms_ at sampling point S1 was 12.5−50.6 dB higher than at sampling point Z1. The porpoise hearing threshold was higher than the 1/3-octave SPL_rms_ at sampling point Z1 in 18.0−39.5 kHz, indicating that the finless porpoise could not hear the underwater noise in this frequency range. This was due to the distance between the transient holding pens and the sound source, so the sound propagation loss caused the SPL at the transient holding pens to decrease. The 1/3-octave SPL_rms_ values at Z1 at <18.0 kHz and 39.5−60.0 kHz were slightly higher than the porpoise hearing threshold curve, so they could perceive the underwater noise in these two frequency ranges.

### 3.4. Results of Behavioral Observations

#### 3.4.1. Respiration Rate

**Figure 7:**
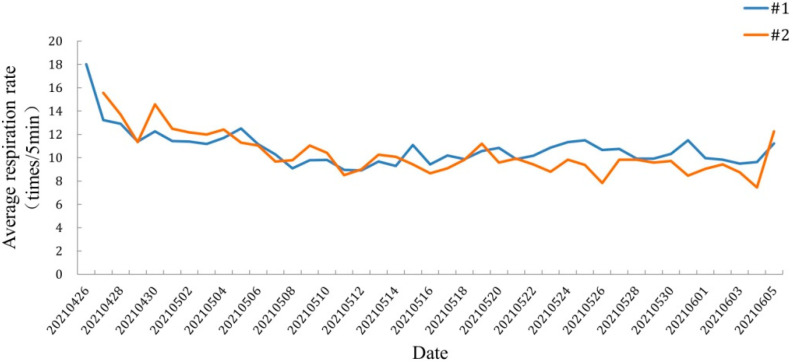
The change curve of respiration rate of six finless porpoises during transient captivity in the holding pens.

Figure 7 shows the average respiration rate of the six Yangtze finless porpoises during the transient captivity. When the finless porpoises were placed in the holding pens, the average respiration rate was 19 times/5min. After about 10 days, the average respiration rate was about 10 times/5min. It was observed that the finless porpoises were swimming stably and basically adapted to the holding pens environment. 

#### 3.4.2. Food Consumption

All the Yangtze finless porpoises did not eat during the first several feeding times. The finless porpoises in holding pen #1 started to eat on the third day, and the finless porpoises in holding pen #2 started to eat on the second day. On the sixth day, May 1 2021, all the finless porpoises had started to eat, and their food consumption was gradually increasing. By May 16, the food consumption of finless porpoises was 3.5 kg per individual each day, and by May 22, the food consumption was up to about 7 kg per individual each day. It was also observed that some small wild fish entered the holding pens and were foraged by the finless porpoises during non-feeding periods, especially at night.

## 4. Discussion

### 4.1. Effects of Noise from the Water Intake Pontoon on the Behavior of Porpoises Temporarily Reared in the Holding Pens

Noise is an acoustic stimulus, and the magnitude of noise induces different behavioral responses in Yangtze finless porpoises [24]. Underwater noise generated by the water intake pontoon had propagation loss before reaching the holding pens. The SL of underwater noise generated by the motor of the water intake pontoon during operation was greater than 149.5 dB (Table 1). The SPL_rms_ value at sampling point Z1 of the transient holding pens was 108.4 dB. As shown in Figure 6, the 1/3-octave SPL_rms_ at the holding pens was mostly lower than the hearing threshold curve of Yangtze finless porpoises. The SPL around 107 dB was thought comfortable for harbor porpoises, which have similar hearing abilities to Yangtze finless porpoises [25]. In addition, Kastelein et al. (2008) discovered that continuous noise is much less likely to have behavioral impacts than intermittent noise [26]. The underwater noise generated by the water intake pontoon is continuous noise. Thus, the possible impact is further reduced. Respiration rates, swimming behaviors, and food consumption are possible indicators to assess the stress and adaptation of porpoises [25,26,27]. In this study, no behaviors reflecting obvious discomfort were observed during the temporary feeding period, and no abnormalities in feeding, breathing, or swimming behavior were observed. Therefore, we considered that the Yangtze finless porpoises in the holding pens can adapt to the continuous noise from the water intake pontoon.

### 4.2. Influence of Water Intake Pontoon Noise on Porpoise Vocalization 

Yangtze finless porpoises use high-frequency echolocation signals to detect, navigate, and forage [28]. The center frequency of the echolocation signal of porpoises in the transient holding pens at the TIAN-E-ZHOU old river course was 128 kHz, and the apparent sound source level was 180 ± 4 dB [29]. Compared with Figure 4, the underwater noise SPL at the holding pens was less than 106.0 dB, and the SPL at Z1, the nearest sampling point to the sound source, was less than 143.5 dB. The overall SPL_rms_ curves at all sampling points decreased gradually as the frequency increased, and was much lower than the source levels (SLs) of the Yangtze finless porpoise echolocation calls. Although the noise at high frequencies could not be estimated since the acoustic recorder did not collect signals higher than 60 kHz, considering that the water intake pontoon does not have high-frequency vibration sources, we considered that the underwater noise at the holding pens would not affect the porpoise echolocation signal.

In addition, transient and captive Yangtze finless porpoises emit continuous low-frequency (generally below 15 kHz with a duration from 300 to 600 ms) acoustic signals [30]. These continuous calls were considered as functions for communication, and recorded when the porpoises were cruising alone or in pods, detecting objects, stimulating the genitals and attempting to mate, during which the frequencies were 1.66 ± 0.35 kHz and the durations were 434.5 ± 225.94 ms [31]. Newborn porpoises in captivity emit contact calls in the range of 2 kHz to 3 kHz, with an SPL of about 130−134 dB. They can emit high-frequency acoustic signals greater than 100 kHz after about 20 days of life, with SPLs of these high-frequency signals at about 150 dB [32]. When juvenile porpoises are around 100 days old, they become more proficient in producing high-frequency acoustic signals [33]. Thus, until then, the juveniles may only rely on low-frequency acoustic signals to communicate with their mothers. The underwater noise at Z1 at the holding pens was greater than 66.1 dB and the frequency range was less than 15 kHz. The SPL was greater than 71.7 dB and the frequency range was 1–3 kHz (Figure 4). The low-frequency acoustic signal propagated in the water with a slower decay rate and wider influence. Because Yangtze finless porpoises can use low-frequency acoustic signals to communicate with each other [30,31], the potential impact of pontoon noise on communication calls, and therefore important social behaviors, is more likely to occur. Therefore, it is thought that underwater noise generated by the water intake pontoon propagating to the holding pens may interfere with porpoise communications and with further effects on communication between mothers and offspring. However, due to the limited space of the holding pens, if the mother and the offspring are frightened and scattered, they will quickly resume contact with each other without harm to the affected juveniles. The low-frequency signals at the holding pens would have little effect on adult porpoises because they are skilled in using high-frequency acoustic signals that are not easily masked.

### 4.3. Influence of Noise from the Water Intake Pontoon on Porpoise Hearing 

High-intensity, long-duration underwater noise environments may lead to temporary threshold shifts (TTS) or even permanent threshold shifts (PTS) in Yangtze finless porpoises. Previous studies have reported that when they were exposed to single-frequency noise at 32 kHz, 140 dB, and 3 min duration, it resulted in a TTS of up to 25 dB to the 45 kHz test signal and required 18 min to recover hearing ability. When a porpoise was exposed to single-frequency noise at 32, 64, and 128 kHz with an intensity of 150 dB and a duration of 1 min, its hearing threshold increased by 30, 33, and 17 dB across the three sets of frequencies, requiring greater than 30, 7, and 10 min, respectively, to regain hearing ability. When the exposure time was extended to 3 min with the same intensity, the hearing thresholds increased by 30, 27.5, and 20 dB at 32, 64, and 128 kHz, and required more than 100, 8, and 10 min to regain hearing ability. The comparison shows that with increasing exposure time, the hearing threshold of porpoises increases at different frequencies, and it takes them longer to recover hearing ability, especially at the low frequency of 32 kHz [10].

The 1/3-octave SPL_rms_ values at most frequencies collected in this experiment did not exceed 130 dB. The 1/3-octave SPL_rms_ values at each frequency in the transient holding pens were less than 95.3 dB, and the SEL was 115.4 dB (Figure 6). Therefore, the Yangtze finless porpoises in transient holding pens would not have experienced temporary hearing threshold shifts. 

## 5. Conclusions

From our findings, it can be concluded that underwater noise generated by the water intake pontoon would not interfere with the high-frequency echolocation signal of the Yangtze finless porpoise. There may have been some interference effects due to the low-frequency acoustic signal, which was more likely to affect juvenile finless porpoises than adults. However, due to the limited space in the transient holding pens, such effects could be quickly compensated. The underwater noise of the water intake pontoon was not high enough to result in temporary hearing threshold shifts in the porpoises. In addition, by observing the feeding, breathing, and swimming behaviors of porpoises, it can be inferred that the Yangtze finless porpoise can adapt to the underwater acoustic environment in transient holding pens. 

However, it should be kept in mind that this study did not overlap with the best range of hearing of the Yangtze finless porpoise, and that high-frequency (60−180kHz) noise should be further measured.

## Figures and Tables

**Table 1:** Broadband noise at each sampling point and estimated source levels.

Sampling Point	Z1	S1	S2	S3	S4
SPL_rms_/dB re 1 μPa	108.4 ± 1.5	142.0 ± 0.2	121.8 ± 0.6	130.7 ± 0.3	132.8 ± 0.6
SEL/dB re 1 μPa^2^·s	115.4 ± 0.6	*	*	*	*
SL/dB re 1 μPa	*	149.5 ± 0.2			

Notes: * means no data.

## Data Availability

The authors declare that the data supporting the findings of this study are available within the article and its supplementary information files, or are available from the corresponding authors upon request.
